# Enhancing a Willingness to Respond to Disasters and Public Health Emergencies Among Health Care Workers, Using mHealth Intervention: A Multidisciplinary Approach

**DOI:** 10.1017/dmp.2023.129

**Published:** 2023-07-21

**Authors:** Amber Mehmood, Daniel J. Barnett, Bee-Ah Kang, Ume-e-Aiman Chhipa, Nargis Asad, Badar Afzal, Junaid A. Razzak

**Affiliations:** 1 Department of Public Health, University of South Florida College of Public Health, Tampa, FL, USA; 2 Department of Environmental Health & Engineering, Johns Hopkins Bloomberg School of Public Health, Baltimore, MD, USA; 3 Department of Health, Behavior and Society, Johns Hopkins Bloomberg School of Public Health, Baltimore, MD, USA; 4 Center of Excellence for Trauma and Emergency, Aga Khan University, Karachi, Pakistan; 5 Department of Psychiatry, Medical College, Aga Khan University, Karachi, Pakistan; 6 Department of Emergency Medicine, Aga Khan University, Karachi, Pakistan; 7 Department of Emergency Medicine, Weill Cornell Medical College, New York, NY, USA

**Keywords:** Disaster preparedness, willingness-to-respond, mHealth, healthcare workers, self-efficacy

## Abstract

Health care workers (HCWs) are increasingly faced with the continuous threat of confronting acute disasters, extreme weather-related events, and protracted public health emergencies. One of the major factors that determines emergency-department-based HCWs’ willingness to respond during public health emergencies and disasters is self-efficacy. Despite increased public awareness of the threat of disasters and heightened possibility of future public health emergencies, the emphasis on preparing the health care workforce for such disasters is inadequate in low-and-middle-income countries (LMICs). Interventions for boosting self-efficacy and response willingness in public health emergencies and disasters have yet to be implemented or examined among emergency HCWs in LMICs. Mobile health (mHealth) technology seems to be a promising platform for such interventions, especially in a resource-constrained setting. This paper introduces an mHealth-focused project that demonstrates a model of multi-institutional and multidisciplinary collaboration for research and training to enhance disaster response willingness among emergency department workers in Pakistan.

Health care workers (HCWs) are increasingly faced with the continuous threat of confronting acute disasters, extreme weather-related events, and protracted public health emergencies.^1,2^ The scale of mass casualty incidents with terrorist attacks and mass shootings, magnitude of natural disasters with widespread societal and infrastructure damages, and complexity of public health emergencies such as coronavirus disease (COVID-19) have profoundly changed HCWs’ perspective of disaster preparedness.^3^ A willingness to respond (WTR), which refers to the attitudinal dimension of health crisis management, in definitional and practical contrast with “ability,” which comprises knowledge and skills, is an indispensable element of effective health system functioning in public health emergencies and disasters.^4–6^ Health system surge capacity challenges focus mostly on “space” and “stuff,” and there has been less attention paid to staff in the context of the HCWs and their willingness to report to work during disasters and public health emergencies such as the COVID-19 pandemic.^7,8^ WTR deficits among emergency department (ED)-based HCWs represent a critical stress-point for local, regional, and global health security.^9^ This is in contrast to emergency volunteering situations where volunteers operate in a free and gratuitous manner to the needs of beneficiaries and contribute to the attainment of common good out of their own will and conviction.

WTR is conceptually and operationally distinct from knowledge and skills acquired through continuous professional development courses and activities.^10^ In the United States, where there are numerous guidelines for hospital emergency operations plans by several agencies, including the Department of Health and Human Services, the American College of Emergency Physicians, and the Joint Commission on Accreditation of Healthcare Organizations, the willingness of emergency HCWs to report for duty in the event of a disaster varies considerably depending upon the nature of the disaster.^11,12^ In low-and-middle-income countries (LMICs), deficits in health care workers’ WTR represent a pronounced vulnerability to sustainable provision of health services in an ever-broadening milieu of emergent public health threats.^13–15^ The ED is the gateway of the health care system in many LMICs such as Pakistan.^16^ It is a common assumption that during a disaster, health care facilities would have adequate and appropriate staff and resources to care for the injured, acutely ill, and their families and would also provide appropriate care to others. In real life, whether an ED HCW would report to or remain at work when a disaster occured depends upon their personal preparedness, dependent care, and concerns for personal and family safety.^14^

One of the major factors that determines ED-based HCWs’ WTR during public health emergencies and disasters is self-efficacy. Bandura defined self-efficacy as “the belief in one’s capabilities to organize and execute the courses of action required to manage prospective situations.”^17^ Self-efficacy is known to have considerable potential explanatory power over behaviors such as self-regulation, achievement striving, coping, choice of career opportunities, and career competency.^18^ Additionally, self-efficacy is a moderator of sensitivity, interpersonal communication, and has been linked to teamwork performance, which is a key in an effective and well-coordinated disaster response.^19,20^

Despite increased public awareness of the threat of disasters and heightened possibility of future public health emergencies, the emphasis on preparing the health care workforce for such disasters is inadequate and substandard in LMICs.^3,15,21,22^ Additionally, most of the preparedness activities revolve around system preparedness rather than individuals.^23,24^ While several investigators have tried to understand the barriers and facilitators to WTR, a key limitation of existing research is lack of approaches to test new models of capacity development among LMIC- based frontline HCWs focusing on self-efficacy and WTR in disasters.^21,25,26^ The current forms of interventions to enhance WTR have certain limitations. First, they have exclusively been implemented to date in high-income settings; second, they have been in-person training-based only; and third, these capacity development activities tend to be time- and labor-intensive for busy HCWs—entailing several hours of training.^27^ Interventions for boosting self-efficacy and response willingness in public health emergencies and disasters are yet to be implemented or examined among emergency HCWs in LMICs.^28^

Technological advances have transformed the everyday lives of billions around the globe. Mobile technology use has rapidly increased from 23/100 persons with mobile cellular subscriptions in 2005 to **110/100** persons in 2021.^29^ Mobile health (mHealth) technology is considered a particularly promising platform in resource-constrained settings for reimagining health care service capacity-building efforts.^30,31^ It, therefore, seems logical to use this platform for capacity development activities among busy HCWs of LMICs whose formal training opportunities might be constrained due to time, cost, and lack of training material tailored to their needs.^32,33^ WTR capacity- building in some of these contexts is a novel innovation, and one which must be developed and supported further.^30^ Furthermore, participatory approaches in technology and interface design set into motion a chain of trust that yields promising results in mHealth research.^34^ Incorporating the learner’s inputs would not only help identify gaps in WTR but also strengthen the user-centered design process of an information-seeking mHealth application.

The iRise (intervention for Response in significant emergencies) is a novel mHealth intervention designed to strengthen LMIC-based HCWs’ self-efficacy and increase their WTR to public health emergencies, including pandemics, and disasters. The iRise would be an interactive, mobile technology-based, training program that would convey the importance of personal preparedness and related concepts so that workers would be better able to fulfill their emergency response roles if asked, or required, by their hospital. In its proposed format, it would be an online digital app available on mobile phone app store for free download to the study participants. The content, which is currently in the English language, covers several disaster scenarios such as extreme weather events, pandemic influenza, and radiological dispersal device emergencies, and blends scenario-based didactic content with individualized interactive activities to boost hospital-based workers’ self-efficacy and related WTR in a contextualized manner. The learning modules would provide important strategies, coping mechanisms, and self-care tips to help responders prepare themselves in the event of a public health emergency or disaster.

The results of the formative research that includes surveys and in-depth interviews, pilot test, and asssesment of product performace will inform the app refinement and potential for scalability, especially in other languages. The first real-world trial of the app would analyze participant feedback on the time to adopt new technology, content organization, ease of use, satisfaction with the digital platform and interface, and ineffectiveness of the technology. Using a checklist to measure user satisfaction, time efficiency, and readiness to release or deploy, further refinements will be made to add new features or changes in the content. This pilot test will help finalize the content, and the platform will be further evaluated for effectiveness in a randomized trial in the next phase of the research, after which it will be made available for general public through mobile phone app stores.

In this context, the aims of this paper are to (1) discuss the theoretical underpinnings of the iRise that address the WTR deficits among HCWs; (2) illustrate the importance of involving ED HCWs in designing an mHealth intervention to address health system surge capacity challenges for disaster response; and to (3) discuss how interdisciplinary and multi-institutional approach could be integrated into mHealth research and disaster response capacity development in LMICs. This project will be conducted in Pakistan with completion of formative research, development, and pilot test of iRise between 2021 and 2023.

## Project Setting

Pakistan is the sixth most populous country in the world, and is one of the global hotspots of geological and hydrometeorological disasters. Karachi is its most populous city and financial hub of the country that has seen a number of natural and manmade disasters in the recent years, including storms and flash floods, extreme heat, structural collapse, and frequent bomb blasts.^35–37^ Karachi has a number of public and private tertiary care teaching hospitals with EDs caring for hundreds of patients every day.^16^

WTR is an acute issue for ED-based HCWs. A shortage of HCWs in EDs has not only adversely affected public health emergencies such as COVID-19 recently, but also has significantly impacted care delivery in mass casualty incidents and weather emergencies.^14,38–40^ In Pakistan, the EDs serve as a safety net for patients who in the absence of an effective primary health care system, seek emergency rooms care with communicable diseases, complications of chronic health conditions, and complications of pregnancy and labor.^16^ In all circumstances, ED-based HCWs play a very important role in and are potentially exposed to a variety of high-stress situations, including everyday life-threatening emergencies, mass casualty incidents, and disaster response.^40^

Our project is based in 2 Pakistani hospitals, with an aim to build capacity and enhancement of WTR among busy HCWs: (1) Jinnah Postgraduate Medical Centre (JPMC) is the largest government-run academic medical center in Karachi, with an extremely busy emergency department catering to trauma, disasters, and other life-threatening emergencies; and (2) Aga Khan University Hospital (AKUH) is a leading health research institution in Karachi, with the nation’s first academic department of Emergency Medicine. The department also has the largest emergency medicine training program in the country.

## Discussion

### iRise’ and Its Theoretical Foundations in Behavioral Science Framework for Strengthening Self-Efficacy and Response Willingness

**Figure 1.**
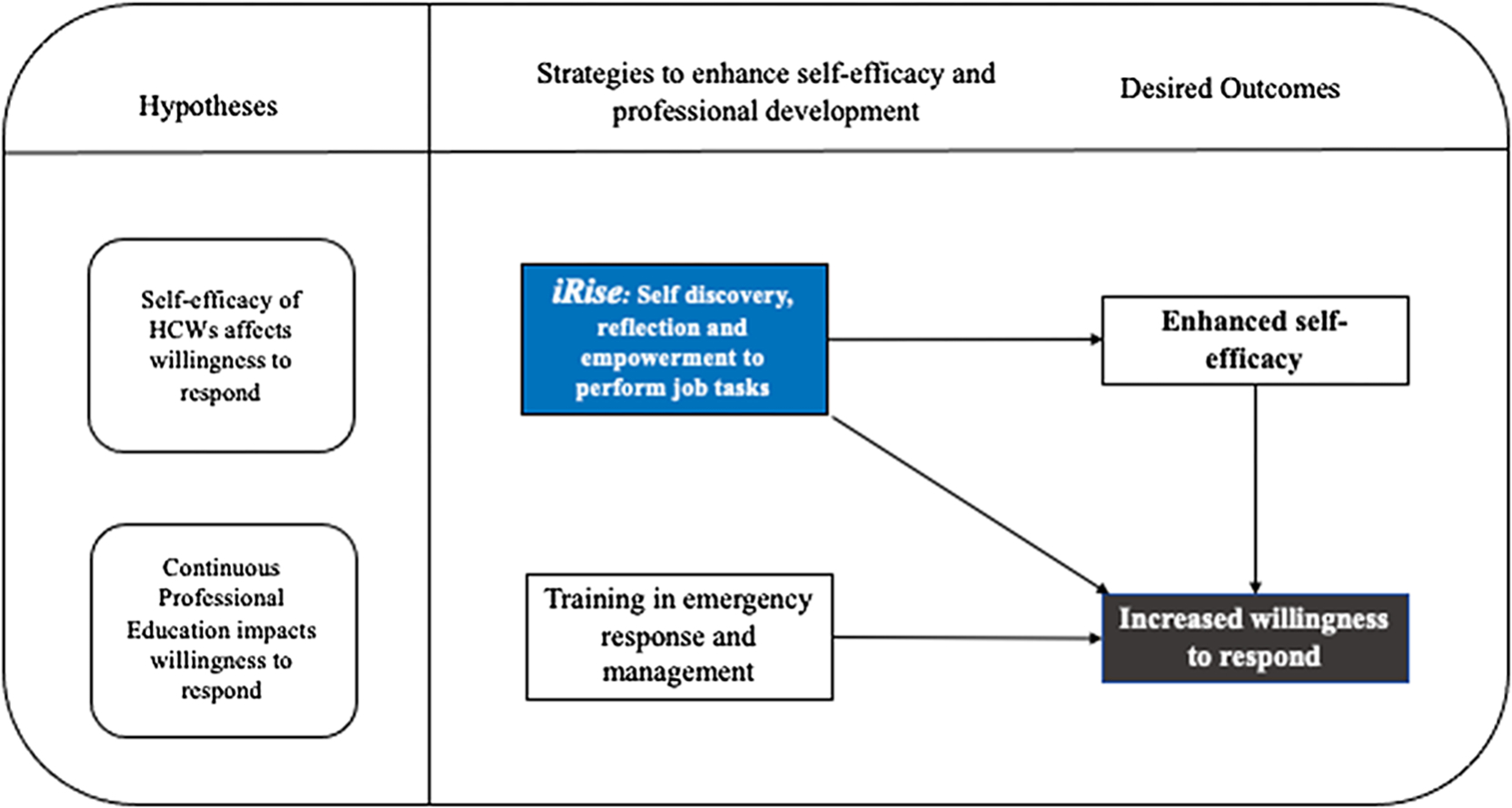
Conceptual overview of mHealth intervention *iRise* on HCWs’ willingness to respond.

Traditional approaches to improve care delivery and increase job-related competencies are geared to increase HCWs’ knowledge and skills that would provide them with the necessary understanding to respond to emergencies. Theoretically, formal training such as those offered through continuous professional development programs for HCWs would positively impact performance of job-related tasks, which in turn would increase WTR in routine and public health emergencies.^41^ However, our hypotheses (Figure 1) are rooted in previously published research that WTR is conceptually and operationally distinct from knowledge and skills acquired through continuous professional development courses and activities,^42^ and that our mHealth intervention, iRise, would enhance LMIC-based frontline HCWs’ perceived self-efficacy and WTR in public health emergencies and disasters.

The iRise application is based on the Extended Parallel Process Model (EPPM) of risk perception that has been used in several contexts to improve self-efficacy and WTR. The EPPM is a multiculturally validated framework that allows an understanding of the adaptive behavior of individuals in the face of uncertain risk. First described by Kim Witte in 1992, the EPPM proposes that threat initiates and motivates message processing in a way that it enhances self-efficacy, response efficacy, and motivates the individual or group of people to control danger by bringing about adaptive changes.^43^ Threat control processes are primarily cognitive processes where individuals evaluate their susceptibility to the threat, the severity of the threat, their ability to perform the recommended response (perceived self-efficacy), and the effectiveness of the recommended response (perceived response efficacy). In order to be effective, messages must contain 2 parts: threat and efficacy, which would be sequentially appraised by the message recipients. Increased perception of threat amplifies the motivation to protection that in turn causes attitude, intention, or behavior changes (ie, adaptive responses) among individuals and group of persons potentially exposed to the hazard of significant severity.

This model has been previously applied to examine the relative influences of perceived threat and efficacy on public health workers’ response willingness to pandemic influenza, workplace safety communication, and, recently, COVID-19 preventive behaviors among dental health care providers, and so forth.^26,41,42,44^ Because of the nature of their job, ED-based HCWs are routinely exposed to potential risk of exposure to biological hazards such as those transmitted through blood, saliva, body fluids, and respiratory secretions, injuries and violence, as well as mental and physical burn-out. Disaster preparedness and response with increasing frequency and of uncertain magnitude pose a continuous risk, and appropriate messaging that deals with threat and efficacy at personal level could potentially be the catalyst to initiate adaptive responses that would eventually increase WTR in emergencies and disaster instead of induce fear and helplessness. This approach is crucial for 2 reasons: (1) maximizing self-efficacy and avoiding unintended consequences such as fear, anxiety, and apprehension toward disaster-related duties; and (2) building HCWs’ resilience in preventing or counteracting the perceived threat that would strengthen health system surge capacity in the long run.

The training and capacity development of the ED HCWs draws upon our previously tested and validated Hospital-Based Infrastructure Training (H-BIT) program.^45^ H-BIT was originally developed and piloted in US hospital settings, with the intended outcome of boosting hospital workers’ WTR to disasters. H-BIT is a threat- and efficacy-focused curriculum based on the EPPM. Prior relevant EPPM-based research on WTR, which informed the original development of H-BIT, points to WTR as a disaster scenario category-specific phenomenon, for which self-efficacy is a leading positive modifier that can be enhanced through training.^42,46^ The curriculum itself was originally informed by an EPPM-based research curriculum—the Public Health Infrastructure Training (PHIT)—which was found to increase WTR to high-dread disaster scenarios among US health department workers.^45^ Through its rigorous incorporation of EPPM, H-BIT’s aim is to boost hospital-based workers’ self-efficacy, and related WTR, toward a variety of representative disaster scenarios, including extreme weather events, pandemic influenza, and radiological dispersal device emergencies.^26^ H-BIT incorporates adult learning theory and blends scenario-based didactic content with individualized interactive activities of research-identified relevance to WTR, including, for example, mapping out multiple routes to one’s worksite as part of disaster-response contingency planning. The EPPM has been validated across multiple countries and contexts,^41^ thus facilitating H-BIT’s adaptability to LMIC settings, its customizability for emergency medicine-based HCWs, and its suitability for mHealth curricular integration.

### The Importance of Involving Emergency Department HCWs in Designing an mHealth Intervention to Address Self-Efficacy for Disaster Response

The formative research for developing this intervention is built on the foundations of evidence informed approach and community-based participatory research. The intervention hinges on promoting and enhancing self-efficacy among HCWs. This concept implies that the intervention will build upon the baseline self-efficacy of HCWs. In the first phase of the project, the baseline self-efficacy will be assessed through a cross-sectional survey of health care workers at 2 leading emergency departments in Pakistan—AKUH and JPMC. The survey participants, including attending physicians, nurses, technicians, and physicians in training—will be queried about 3 representative scenarios within the all-hazards spectrum: weather disaster; pandemic; and manmade radiological events (ie, radiological “dirty” bombs)—indicating their attitudes and beliefs based on a series of 20 construct statements relevant to emergency response risk perception.

Involving ED-based HCWs in the intervention design is similar to the community-based participatory research^47^ that is based on the following core principles: (1) It is participatory and requires cooperation between the research team and subjects or target population; (2) there is a mutual exchange of knowledge and information; (3) there is empowerment and ownership of the intervention due to shared decision-making; (4) it builds upon the strengths of the target community, therefore, promotes sustainability; (5) the implementation of the intervention is based on contextual findings; (6) the context recognizes subject in a social setting instead of just physical location; and (7) there is long-term relationship building among the stakeholders. All these principles are vital in developing an intervention for a subset of HCWs whose insights, experiences, and expectations of disaster preparedness and response would be invaluable for tailoring the content, delivery, and design of instructional material.

The implementation of people-centered capacity development requires strategies that respond to local conditions and contexts and involve local stakeholders. Co-design is a process in which targeted end users and other relevant stakeholders form a partnership with researchers and work together on all aspects of mHealth intervention development, from needs assessment to content development, pilot-testing, and dissemination.^48^ This approach has specific benefits in the context of designing an mHealth intervention to address self-efficacy for disaster response in an LMIC-based setting. First, it would ensure that the adaptation is relevant to the context of low-resource settings and the hazard/risk profile of LMICs; second, an understanding of existing challenges, coping mechanisms, and self-care strategies would help produce culturally nuanced and contextually feasible content related to self-efficacy and WTR; third, gathering information regarding applicability of a mobile application (instructional design, user interface) and training strategy (competencies, number and duration of modules, assessment) would help design an application that suits the highly dynamic and stressful context of ED-based HCWs.

The development of the intervention would be based on surveys and in-depth interviews with a representative sample of HCWs from each cadre of ED. Subsequently, a selected group of ED-based HCWs at AKUH and JPMC will participate in a pilot test of the mobile application to gauge user acceptance and evaluation of the product performance in the real world. This strategy would include task and survey distributions to guide HCWs in their engagement with the app and allow each user to discover product features. It would help establish the time duration to adopt new technology, perceived usefulness and satisfaction, organization of content, ease of use, mitigation of challenges in integrating more technology into an already complicated technological environment, and effectiveness of the technology. This pilot test would help make further refinements, add new features, or make changes in the content.

### Applying Interdisciplinary and Multi-Institutional Approach for Integrating mHealth Research and Capacity Development in LMICs

Complex public health challenges require an interdisciplinary collaborative approach. One of the strengths of this research project resides with its multi-institutional team. The research team and partners bring extensive and complementary combined expertise in public health emergency and disaster response, mental health, curriculum and instructional design, and mHealth-based research. Team membership from Johns Hopkins University (JHU), University of South Florida (USF), Weill Cornell, AKUH, and JPMC has extensive expertise in research on public health emergency preparedness and response systems, including disaster preparedness and planning, humanitarian and mass casualty response management, epidemiology, mHealth capacity development, as well as psychological and behavioral response modifiers in crisis situations, both in high- and low-income countries. Our team features a combination of critical knowledge and expertise, which are both global as well as uniquely context specific. This project also provides an invaluable opportunity of combining research with capacity development and knowledge exchange. The value of integrating capacity development in research projects has been emphasized in the context of increasing popularity of mHealth interventions in LMICs.^49^ Formal and informal training of the project team, resources sharing, networking, joint data collection, and analysis across individual, institutional, and project levels are some strategies that reinforce the essense of cross-cultural learning and sustainability in such research endeavors.

### Limitations

The iRise is expected to directly influence the self-discovery process, including reflection, coping strategies, and self-regulated learning, which are the behavioral aspects of personal competence and key to enhancing self-efficacy. However, there are some challenges and opportunities when using mHealth-based interventions to improve self efficacy and response capacity. This paper is not examining emergency volunteering behaviors, but rather it is examining fulfilling disaster response job roles for non-volunteers. Improving self-efficacy may not always lead to actual behavior but is a strong predictor of behavior according to Bandura.^18^ A behavioral intervention is limited in shifting behaviors in a sustainable way unless accompanied by proper institutional support and policy change. We expect evidence from our study would inform the development of organizational-level support and facilitate discussion among hospital leadership and policy makers. Our mHealth training does not address participants’ questions or concerns in real time unlike in-person lectures. This limitation may be mitigated through collecting user feedback and evaluation of the mHealth app and responding to the users’ questions through a Q&A section built into the mHealth application. Additionally, because Pakistan is frequently experiencing various disaster scenarios, regular updates of the mHealth application’s educational content may be necessary, reflecting up-to-date circumstances, once the pilot test is completed. The iteration of the app will be conducted throughout the project duration.

## Conclusions

The knowledge to be gained from this research is of seminal importance for gauging and addressing willingness gaps in LMIC-based frontline health responders toward public health emergencies and disasters. The resulting insights from this study will provide the critical and timely basis for scaled-up, evidence-based, mHealth-based interventions in LMICs, to enhance attitudinally. If found feasible and effective, such a novel mHealth tool could strengthen staffing surge capacity among frontline HCWs and enhance health systems’ functioning in LMICs toward providing a broad array of clinical services in these situational contexts.

Building on the lessons and success of the formative research, capacity development activities, and pilot-testing of the iRise app, 2 additional research projects will be undertaken. First, an effectivesness trial is to be conducted to gauge short-term (1-month), medium-term (6-months), and longer-term (1-year) impacts of this novel iRise mHealth app on LMIC-based HCWs’ self-efficacy and WTR during public health emergencies and disasters; and, second, the mHealth app will be refined to enhance its attitudinal and related impacts on LMIC-based HCWs’ self-efficacy and response willingness.

Meaningful integration of capacity-building efforts into mHealth research is critical if mHealth is to fulfill its potential as a vital component of global disaster preparedness and response. This project demonstrates a model of multi-institutional and multidisciplinary collaboration to advance the sustainable innovation enterprise of research and training for the disaster health workforce.

## Abbreviations:

AKUH: Aga Khan University Hospital
ED: emergency department
EPPM: Extended Parallel Process Model
HCW: health care worker
JPMC: Jinnah Postgraduate Medical Centre
LMIC: low-and-middle-income country
WTR: willingness to respond
